# Community-Acquired Pneumonia Progressing to Necrotizing Pneumonia Due to Congenital Anatomical Abnormalities in the Lung

**DOI:** 10.7759/cureus.26591

**Published:** 2022-07-05

**Authors:** Seyed Mohammad Nahidi, Ubhi Manveer, Danial Sanchez, Luis E Irizarry Nieves, Karthik Seetharam, Parvez Mir

**Affiliations:** 1 Internal Medicine, Wyckoff Heights Medical Center, Brooklyn, USA; 2 Internal Medicine, St. George's University School of Medicine, Grenada, GRD; 3 Pulmonary Critical Care, Internal Medicine, Wyckoff Heights Medical Center, Brooklyn, USA

**Keywords:** pneumonia, bronchoscopy, pulmonary disease, congenital anatomical abnormalities, necrotizing pneumonia

## Abstract

Pneumonia is generally a treatable disease but there are instances when physicians are faced with rare circumstances such as congenital structural abnormalities. Structural abnormalities in the lungs may predispose to pneumonia and other complications. We present a patient with pneumonia, which progressed to necrotizing pneumonia. A diagnostic bronchoscopy was performed and identified multiple accessory lobes in the right lung. Multiple accessory lobes are not easily identifiable by diagnostic imaging such as X-rays or computed tomography scans. As a result, treating pneumonia in patients with such structural anomalies can further complicate management. Currently, there is limited information that correlates pneumonia and accessory lobes with necrotizing pneumonia.

## Introduction

Numerous causative agents may cause pneumonia such as bacterial, viral, or fungal infections. These microorganisms may be acquired through the community, ventilators, or aspiration. Pneumonia is generally a treatable disease but there are instances when physicians are faced with a rare case such as a patient with congenital structural abnormalities. Structural abnormalities in the lungs may increase the risk of pneumonia, complications, and other pulmonary diseases. We present a patient with pneumonia that progressed to necrotizing pneumonia. A diagnostic bronchoscopy was performed, and it identified multiple accessory lobes of the right lung. Multiple accessory lobes are not easily identifiable by diagnostic imaging, such as X-rays or computed tomography (CT) scans. As a result, treating pneumonia in patients with such anatomical structures can be complicated. There is currently limited information that correlates accessory lobes, pneumonia, and necrotizing pneumonia.

## Case presentation

A 77-year-old female, with no past medical history, presented to our institution after being found lying on the ground at her home for an unknown period of time. The sepsis protocol was initiated due to the patient’s tachycardia of 116 beats per min, white blood cell (WBC) count of 37.30 k/ul (normal range (NR): 4.5 - 10.9 k/ul), and lactic acid of 3.7 mmol/L (NR: 0.4 - 2.0 mmol/l).

**Figure 1:**
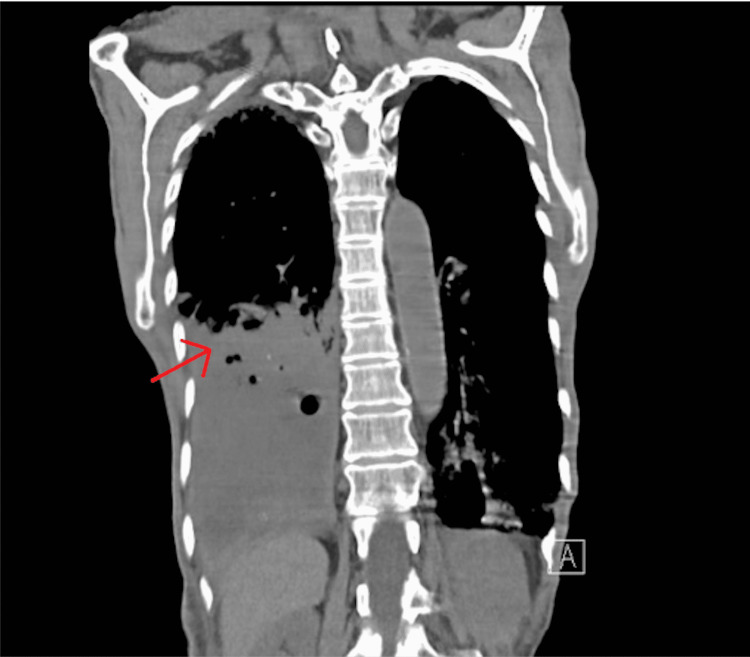
Coronal CT scan without contrast identifying bilateral pulmonary infiltrates with a large air-fluid collection (pointed to by the red arrow) within the right lobe

**Figure 2:**
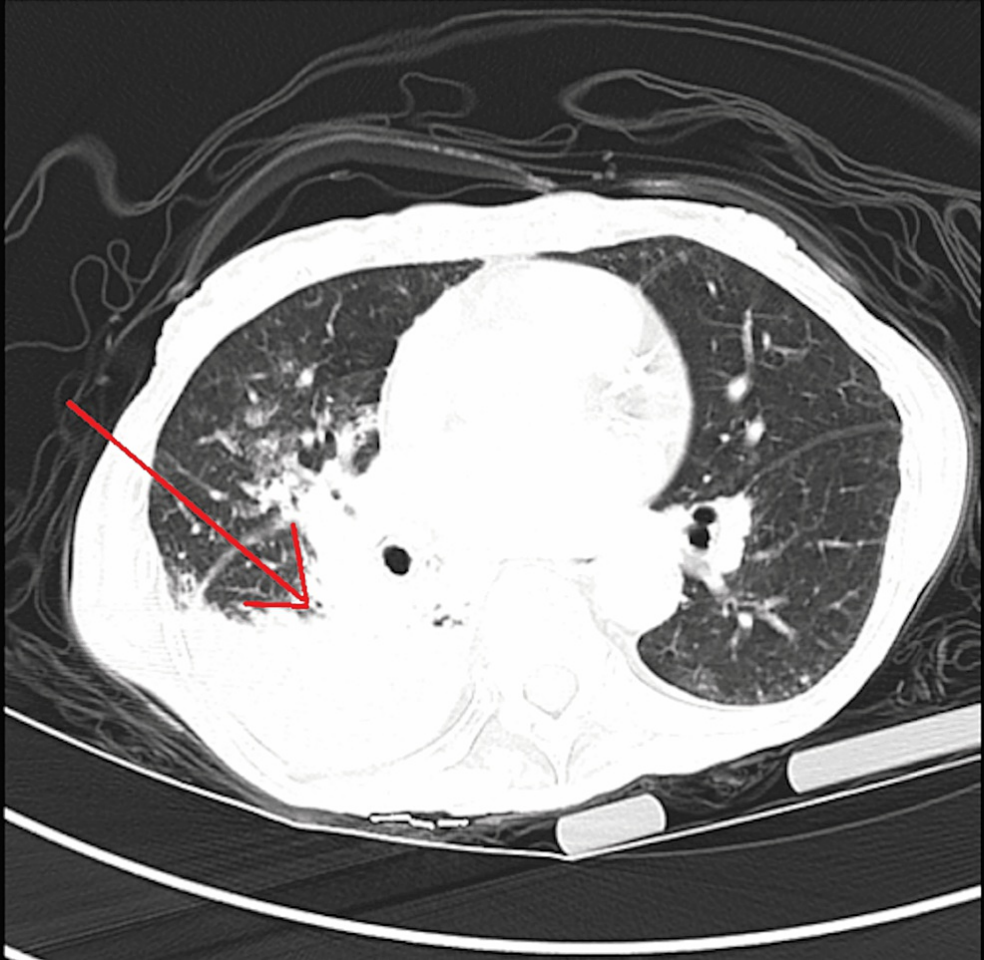
Axial CT scan without contrast identifying bilateral pulmonary infiltrates with a large air-fluid collection (pointed to by the red arrow) within the right lobe

The CT scan of the chest without contrast identified patchy bilateral airspace opacities, predominantly within the right middle lobe and left lower lobe, and large air-fluid collection within the right lower lobe (Figures 1-2). The pulmonologist suspected necrotizing pneumonia and recommended vancomycin, piperacillin/tazobactam, azithromycin, and possible bronchoscopy if there was no clinical improvement.

On hospital Day 6, the patient became severely hypoxic while on a 3-liter nasal cannula and developed hypercapnic hypoxic respiratory failure. On arterial blood gas, the ph was 6.98, partial pressure of carbon dioxide (pCO2) 118 mmHg, partial pressure of oxygen (pO2) 82 mmHg, and bicarbonate (HCO3) 27.8 mmol/l. The patient was intubated and transferred to the intensive care unit (ICU). Fluids and norepinephrine bitartrate were started due to hypotension. The WBC count had trended down to 17 k/ul, however, clinical presentation suggested worsening pneumonia. She was started on meropenem 1 gm per eight hours, linezolid 600 mg twice per day, and doxycycline 100 mg twice per day.

**Figure 3:**
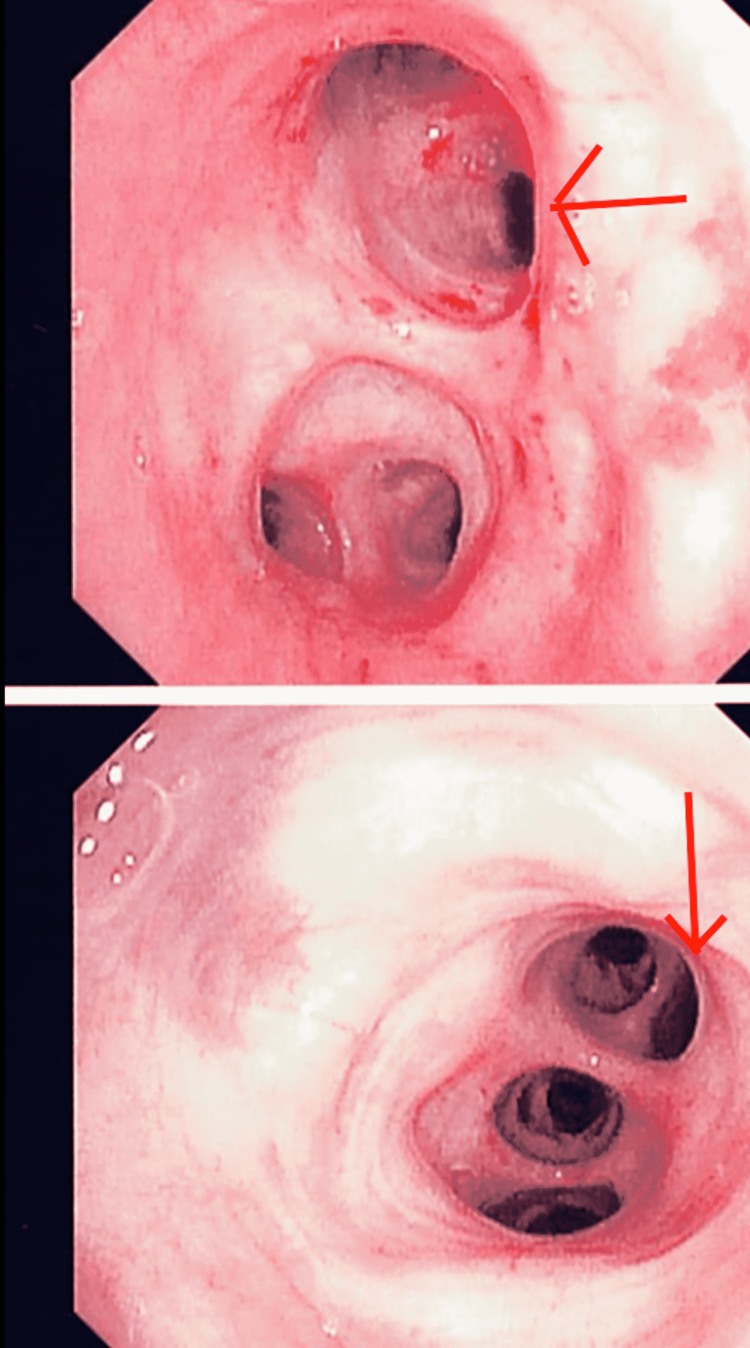
Images from the bronchoscopy identifying accessory lobes The red arrow indicates the accessory lobes. Bronchoscopy confirmed the patient has additional lobes. The top image refers to the right medial lobe of the right lung. The bottom image refers to the bronchus intermedius of the right lung. RML = Right Medial Lobe; BI = Bronchus Intermedius

Due to the worsening clinical presentation, a bronchoscopy was warranted. The bronchoscopy identified multiple accessory lobes predominantly on the right upper and middle lobes and copious purulent secretion in both lungs but predominantly on the right side (Figure 3). The bronchial aspirate grew rare yeast and Strep epidermidis. Fluconazole 100 mg intravenous was added. The blood cultures were negative.

On hospital Day 14, a tracheostomy procedure was performed due to an inability to wean off the ventilator. As per the infectious disease specialist, the antibiotics and antifungals should run for 14 days. On hospital Day 21, the patient still required vasopressor therapy due to persistent hypotension. The patient expired due to cardiopulmonary arrest on hospital Day 25.

## Discussion

A rare, but severe, complication of pneumonia is NP, which presents as a progressive pulmonic illness. In our patient, rare yeast was cultured from the bronchial aspirate, which is an uncommon organism, and it is rare for this pathogen to develop into life-threatening diseases such as in this patient [1]. The mortality associated with community-acquired pneumonia (CAP) requiring an ICU admission has been reported to be 30% [2], with greater mortality in those with NP [3]. Therefore, it is necessary to diagnose patients with pneumonia early during the infectious course and identify any congenital or anatomical anomaly predisposing them to further complications.

Accessory fissures are congenital pulmonary anomalies that result whenever the space between the lobes fails to obliterate during development [4]. As a result, accessory lobes develop secondary to these accessory fissures. A variety of accessory lobes have been reported, with the most common being the azygos lobe and dorsal lobe. Biswas et al. reported the prevalence of these lobes as 2.17% and 1.08%, respectively [5]. Patients with an accessory airway branch have been associated with a 1.64 higher risk of chronic obstructive pulmonary disease (COPD) and bronchitis [6-7].

Accessory lobes are usually not discovered unless complications arise or through an incidental finding. Biswas et al. showed that the majority of the time, an accessory lobe was misdiagnosed as either an abscess or bullae on a chest X-ray [5]. In our case, the accessory lobes were not identified on radiographic imaging but were identified via bronchoscopy. Certain complications may arise through bronchoscopy such as bleeding, collapsed lung, and infections [8]. Additionally, a CT scan of the chest has proven unreliable in detecting accessory fissures and lobes due to its incompleteness, thick CT scan sections, or in an orientation relating to a particular anatomical plane [3]. In an autopsy study, the reported incidence of superior accessory fissures was 5-30% compared to the 3% incidence in high-resolution CT scans [9]. Imaging is very crucial in diagnosing necrotizing pneumonia and understanding the etiology behind it. CT scans have been the most sensitive diagnostic tool for diagnosing necrotizing pneumonia [3].

To our knowledge, this is the first case report discussing the correlation between pulmonary accessory lobes and necrotizing pneumonia. Our case report seeks to shed light on the complications that come with accessory lobes anomalies, and imaging tools utilized in the diagnosis of these accessory lobes to help aid in management.

## Conclusions

However, in instances where patients have rare anatomical abnormalities, treating them may be complicated, and it may lead to fatal outcomes. Congenital structural abnormalities of the lungs are not easily identifiable and may require more aggressive diagnostic testing in order to better characterize the variation in lung anatomy. Once identified, a correlation between abnormal lung anatomy and severe pneumonia complications like necrotizing pneumonia can help guide clinicians in their diagnosis and treatment.
